# Model for utilizing distance learning post COVID-19 using (PACT)™ a cross sectional qualitative study

**DOI:** 10.1186/s12909-020-02311-1

**Published:** 2020-11-02

**Authors:** Samar A. Ahmed, Nagwa N. Hegazy, Hany W. Abdel Malak, W. Cliff Kayser, Noha M. Elrafie, Mohammed Hassanien, Abdulmonem A. Al-Hayani, Sherif A. El Saadany, Abdulrahman O. AI-Youbi, Mohamed H. Shehata

**Affiliations:** 1 Professor of Forensic Medicine, Director Ain shams University Middle East North Africa FAIMER Regional Institute, Ain shams University, Cairo, Egypt; 2 Associate professor of Family Medicine, Director of Medical education and Human Resources Center Faculty of Medicine, Menoufia University, ASU MENA FAIMER Regional Institute 2019 fellow, Menoufia, Egypt; 3 Professor of Anatomy & Embryology at faculty of medicine - ASU & AFCM. Director of online presence at ASU MENA FAIMER, Ain shams University, Cairo, Egypt; 4 Chief Science Officer, SixSeed Partners, Washington, D.C USA; 5 Assistant lecturer of Forensic Medicine and Toxicology, Faculty of Medicine, Ain Shams University, ASU MENA FAIMER Regional Institute 2019 fellow, Cairo, Egypt; 6 King Abdulaziz University, Vice presidency for educational affairs and college of Pharmacy and College of Medicine, Tanta University, Tanta, Egypt; 7 University Vice President for Educational Affairs and Department of Anatomy, Faculty of Medicine, King Abdulaziz University, Jeddah, Saudi Arabia; 8 College of Pharmacy, King Abdulaziz University, Jeddah Saudi Arabia, College of Medicine, Tanta University, Tanta, Egypt; 9 University President and Chemistry Department, Faculty of Science, King Abdulaziz University, Jeddah, Saudi Arabia; 10 Professor of Family Medicine, College of Medicine and Medical Sciences, Arabian Gulf University, Kingdom of Bahrain and Faculty of Medicine, Helwan University, Helwan, Egypt

**Keywords:** Planning, Distance learning, Polarity mapping, COVID

## Abstract

**Background:**

COVID − 19 pandemic pressured medical schools globally to shift to Distance learning (DL) as an alternative way to ensure that the content delivered is satisfactory for student progression.

**Aim of the work:**

This work aims at mapping priorities for post-COVID planning for better balance between distance learning and face to face learning.

**Methods:**

This qualitative study aimed to develop a model for utilizing DL using The Polarity Approach for Continuity and Transformation (PACT)™. A virtual mapping session was held with 79 faculty from 19 countries. They worked in small groups to determine upsides and downsides of face-to-face and DL subsequently. An initial polarity map was generated identifying five tension areas; Faculty, Students, Curriculum, Social aspects and Logistics. A 63-item assessment tool was generated based on this map, piloted and then distributed as a self-administered assessment. The outcomes of this assessment were utilized for another mapping session to discuss warning signs and action steps to maintain upsides and avoid downsides of each pole.

**Results:**

Participants agreed that face-to-face teaching allows them to inspire students and have meaningful connections with them. They also agreed that DL provides a good environment for most students. However, students with financial challenges and special needs may not have equal opportunities to access technology. As regards social issues, participants agreed that face-to-face learning provides a better chance for professionalism through enhanced team-work. Cognitive, communication and clinical skills are best achieved in face-to-face. Participants agreed that logistics for conducting DL are much more complicated when compared to face-to-face learning. Participants identified around 10 warning signs for each method that need to be continuously monitored in order to minimize the drawbacks of over focusing on one pole at the expense of the other. Action steps were determined to ensure optimized use of in either method.

**Conclusion:**

In order to plan for the future, we need to understand the dynamics of education within the context of polarities. Educators need to understand that the choice of DL, although was imposed as a no-alternative solution during the COVID era, yet it has always existed as a possible alternative and will continue to exist after this era. The value of polarity mapping and leveraging allows us to maximize the benefit of each method and guide educators’ decisions to minimize the downsides for the good of the learning process.

## Background

COVID-19 pandemic has and has still yet to continue its dramatic effect on the world and how we function as communities. It led to an extensive disturbance of medical and health professions education and training as well as continuous professional training [1] & [2]. Actions to ensure social distancing have essentially included closure of all medical schools with a sudden shift from the face to face context to the online context for instructors, students and trainees. Colleges and universities all around the globe are working hard to overcome this crisis using different technologies to maximize the learning experience of the students [3].

A large amount of online teaching started in response to this crisis. This took so many formats and used many interfaces in an attempt to deliver content and a false expectation developed that students should take responsibility for their own learning.

Within the unplanned rapid transition, more focus was set on compliance with technical needs and requirements at the expense of student centeredness, engagement and the effectiveness of the educational environment [4].

There is no doubt that distance learning (DL) is extremely vital and is as important as face to face learning. DL provides flexible learning opportunities according to the interests of students and lecturers either in asynchronous or synchronous online learning. On the other hand face-to-face learning demonstrates the interaction with learners, facilitating the convenience of cooperative learning and also the clarity of learning material [5]. Distance learning when managed well, allows for a good learning opportunity where education happens. The main problem that faces distance learning beyond doubt is the extra effort needed to keep the learners from dropping out and losing touch of the required deliverables. Face to face learning on the other hand carries the risk of overwhelming the system with logistic requirements.

Developing and sustaining high-quality education for students is no longer achieved solely in a classroom. A hybrid curriculum is what is needed with a clear distinction of the red flags for each experience [6]. "Distance learning is itself a form of blended learning, using a variety of coordinated and planned modalities and methods to deliver the curriculum and enable students to learn effectively" [7].

Polarity Management, originally developed by Barry Johnson, is a powerful way for individuals, groups and systems to grow in this direction.

A polarity is a pair of interdependent positive concepts that need to work together for sustainable and optimal effectiveness, such as Consistency & Flexibility.

The Polarity Approach to Continuity and Transformation™ provides a 5-Step approach using tools and principles to leverage polarities we live in. In his recent book, “And: Making a Difference by Leveraging Polarity, Paradox or Dilemma Volume One: Foundations” Barry Johnson describes polarities as “interdependent pairs that need each other over time.” [8] Polarities are interdependent pairs that work together over time to achieve a greater purpose shared by both poles of the polarity. One example is inhale and exhale. We don’t choose between one or the other -- we must do both to live. Another example is activity and rest. We do both over time to maintain our mental, physical, emotional and spiritual health. Choosing one pole of a polarity as a “solution” over time, undermines potential for sustainable high performance. Successful leaders, teams, and organizations that make the distinction between problems to solve and polarities to leverage outperform those that don’t.

The Polarity Approach to Continuity and Transformation™ is about Seeing, Mapping and Leveraging polarities that will have the greatest positive impact on teams, and/or in organizations. In this first step you will begin thinking through the polarities that will provide the greatest leverage. You will also explore how polarities may help you better understand the issues and opportunities you’re facing right now – both internally and externally.

In the second step, you’ll build Polarity Maps® for each of the key polarities you identified as being most important to you. Polarity Maps® contain the greater purpose & desired results you’ll achieve from leveraging a Polarity well in addition to the negative results you’ll experience if you don’t.

Polarities can be assessed using low or high-tech methods – they are all designed to be “high touch.”

Regardless of the assessment method you choose, a Polarity perspective drives insights that create a uniquely positive experience and makes much more possible in response to the question, “What can we do to leverage this Polarity better?”

### Leveraging is about getting more with less

The synergistic energy generated in a virtuous cycle can help you achieve your highest aspirations with less effort and sustain these results over time. The development and execution of Action Steps, to go after the upsides of both poles, create synergistic opportunities that is the heartbeat of a virtuous cycle. Monitoring Early Warnings that signal when you are starting to experience downsides due to an over or under focus, protect you from the destructive forces contained in a vicious cycle.

Early Warning Signals can be difficult to identify (late warnings are more obvious) and are almost always a challenge to monitor consistently. These plans you put in place should build on what you’re already doing well, improve on what you want to do better, and provide mechanisms to track your progress and course correct over time.

This work aims at mapping priorities for post-COVID planning for better balance between distance learning and face to face learning.

## Methods

This is a qualitative study utilizing content analysis for deductive analysis. The 5-Steps of the PACT process was followed: Seeing, Mapping, Assessing, Learning, and Leveraging [8].

*Step 1, Seeing*. The team identified the tension in the shift from the pole of face-to-face learning and the embrace of the pole of Distance Learning.

*Step 2, Mapping*. Seeing the polarity in more detail is accomplished by mapping key dimensions of the tension with a diverse group of key stakeholders. Nearly 79 medical educators mainly from Middle East countries included faculty and educational leaders from 19 countries and at different levels of the command chain in educational decisions in their schools. 30% of the attendees were marked as decision makers while the rest were active educators in schools of health professions. Using the Polarity Map, this team mapped the polarity on a virtual platform via Zoom cloud meeting. Attendees were first introduced to the core definitions of terms needed throughout the session, for example distance learning, E-Learning and online learning and the discrepancies between the different terms.

An emphasis was placed on the core definition of distance learning and the overarching definition offered by Grant (2008): Individual study of specially prepared learning materials, usually print and sometimes e-learning, supplemented by integrated learning resources, other learning experiences, including face-to-face teaching and practical experience, feedback on learning and student support [9].

The definition DL adopted was placed in contrast to the technology based education that was the first form that jumped to the minds of respondents. Emphasis was placed on this contrast throughout the data collection activities.

Attendees were then introduced to the concept of polarities and how they can be used to map key dimensions of the tension in the interdependence using a Polarity Map. They were then broken out into four groups using the zoom breakout room function each with an escorting design team member. Each group was given a question from the Polarity Map to respond to:

- *(Group 1) What are the benefits that emerged from the use of distant learning post COVID-19?*
- *(Group 2) What are the benefits of face to face teaching that we realized after we had to experience the post COVID-19 social distancing?*
- *(Group 3) What are the drawbacks that emerged from the use of distant learning in post COVID-19?*
- *(Group 4) What are the drawbacks of face to face teaching that we realized after we had to experience the post COVID-19 social distancing?*

### Data analysis

*The video record was transcripted by the researcher and coded. Results of the mapping were analysed and categorized thematically resulting in When the team analyzed the results of the mapping process on the Polarity Map, they began to notice key category dimensions or key themes of focus in each of the quadrants. They were: Faculty, Students, Curriculum, Social aspects and Logistics.. Under these all the data was accounted for. Two independent researchers were asked to verify the seeming accuracy of the category system and after discussion with them, minor modifications were made.*

Individual Polarity Maps were then developed focusing specifically on those categories areas for face-to-face and distance learning**.**

*Step 3, Assessing.* For each Polarity Map, assessment statements were deducted utilizing the PACT language, and a sixty-three item assessment was generated using a tool developed by Polarity Partnerships to custom polarity-based assessments (the Polarity Assessment) (https://assessmypolarities.com/). A smaller team of 7 medical educators guided the refinement of the assessment item phrasing and language to ensure they corresponded to the desired outcomes. They also piloted the survey before sending it out to the larger population. The assessment was then deployed electronically to staff members in different universities.

*Step 4, Learning.* Results of the assessment were analyzed, and the group made meaning of these results based on the current realities. Using this benchmark data, the group began the process of developing strategies to maximize the benefits and minimize the limitations for each polarity based on the learning from the results. Results of the assessment were represented electronically on the polarity assessment web tool. The results in each quadrant of the map was coded according to the frequency of its occurrence in the results. Results for each of the items were colour coded according to frequency into mild, moderate and severe each with a separate colour (http://assessmypolarities.com/). Items falling in the moderate and severe zone were selected and were placed as focus for the leveraging step.

*Step 5, Leveraging.* In this step the strategy to maximize upside benefits (Action Steps) and minimize downside limitations (Early Warning Signs) are identified to complete the Polarity Map and final step of the 5-Step process. In an online session, the group divided into four sub-groups, each assigned one question for each of the polarities:

- *Group 1: What are the steps you and/or your organization can take to maintain the benefits that emerged from the use of distant learning post COVID-19?*
- *Group 2: What are the steps you and/or your organization can take to maintain the benefits that emerged from the use of face to face learning?*
- *Group 3: What are the signs that you and/or your organization should look for to avoid experiencing the drawbacks that could emerge from the use of distant learning when planning in the future of your education interface?*
- *Group 4: What are the signs that you and/or your organization should look for to avoid experiencing the drawbacks that could emerge from the overuse of face to face learning when planning in the future of your education interface?*

*Participants in this study are faculty from all over the region with a user level capacity of distance learning. Faculty involved in the study had previous knowledge and experience in running distance learning courses but were never subjected to the mandate to transfer all curricula to a distance learning design before the COVID-19 pandemic.*

*Inclusion criteria in the focus group included being faculty of a medical school in the MENA region, with experience in medical education development at the level of the school or the country.*

*The questionnaire was sent to faculty of medical schools in the MENA region at all levels of seniority.*

*Sample size was calculated using the formula:*

Sample Size = [z2 * p(1-p)] / e2 / 1 + [z2 * p(1-p)] / e2 * N]

- *N = population size*
- *z = z-score*
- *e = margin of error*
- *p = standard of deviation*

*Population size was estimated differently for every stage of the data collection at a confidence interval of 95%. For the focus group a purposeful sample was selected to represent the universities in Egypt, where is step 1 the purposive sample was 79 and in step 2 it was 7. The questionnaire respondents sample was calculated as 213.*

The results of this process were collected and shared.

## Results

**Fig. 1:**
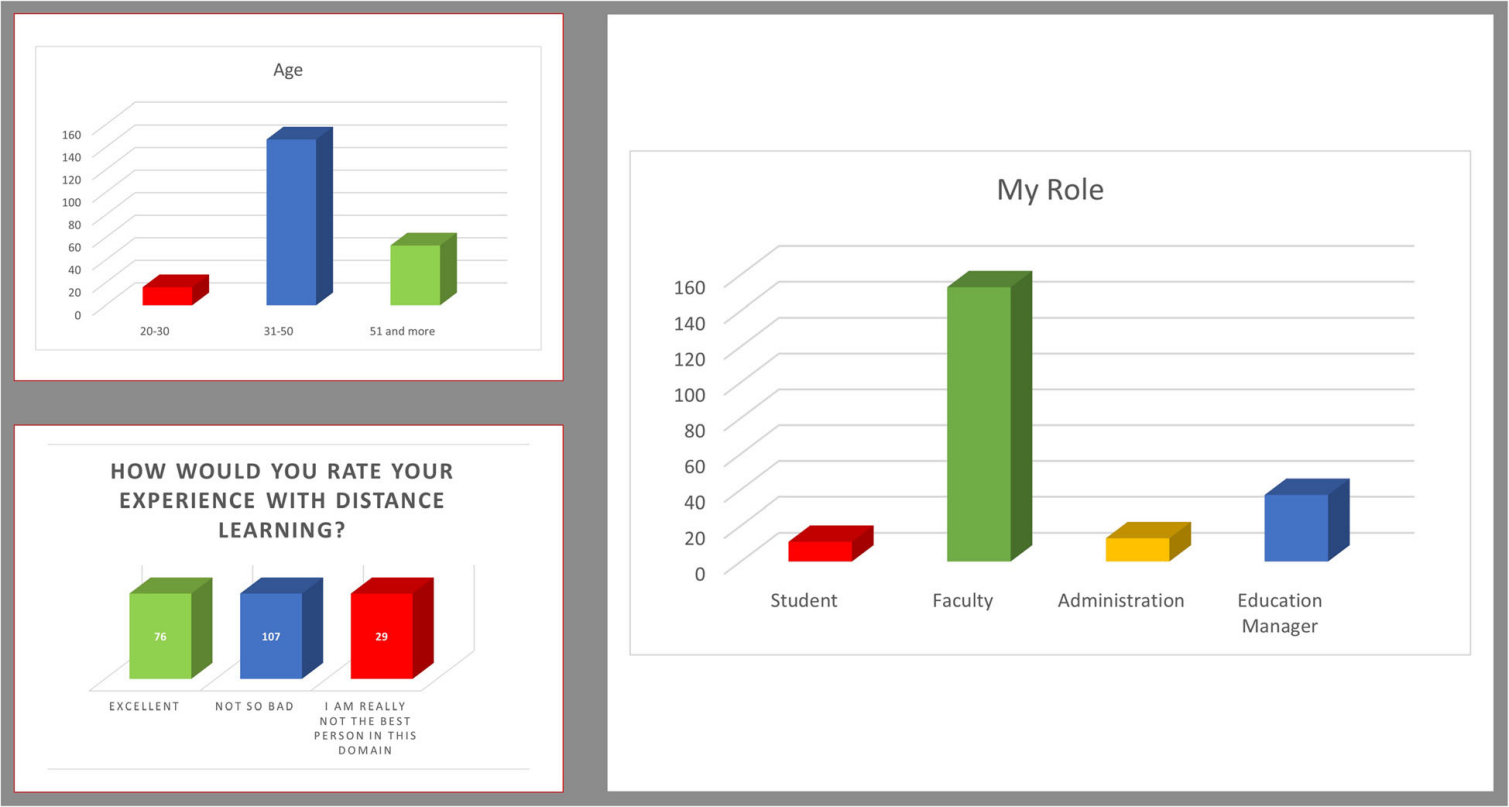
Demographic information of respondents

Almost 70% of the participants were in the age group 31–50 years-old-faculty who rated their experience in technology as not bad (Table 1 & Fig. 1).

**Table 1:** Demographic data of respondents

Demographic	Number
Age	
20–30	2016
31–50	140147
≥ 51	4053
Role	
Student	1011
Faculty	140153
Administration	1013
Education manager	4037
Experience with distance learning	
Excellent	2076
Not so bad	140107
Really the best person in this domain	4029

The resulting information was mapped into five maps each demonstrating one tension area (Teachers, students, Curriculum, Social and Logistics).Results of the assessment are highlighted in each area below:

### First tension area: faculty

**Fig. 2:**
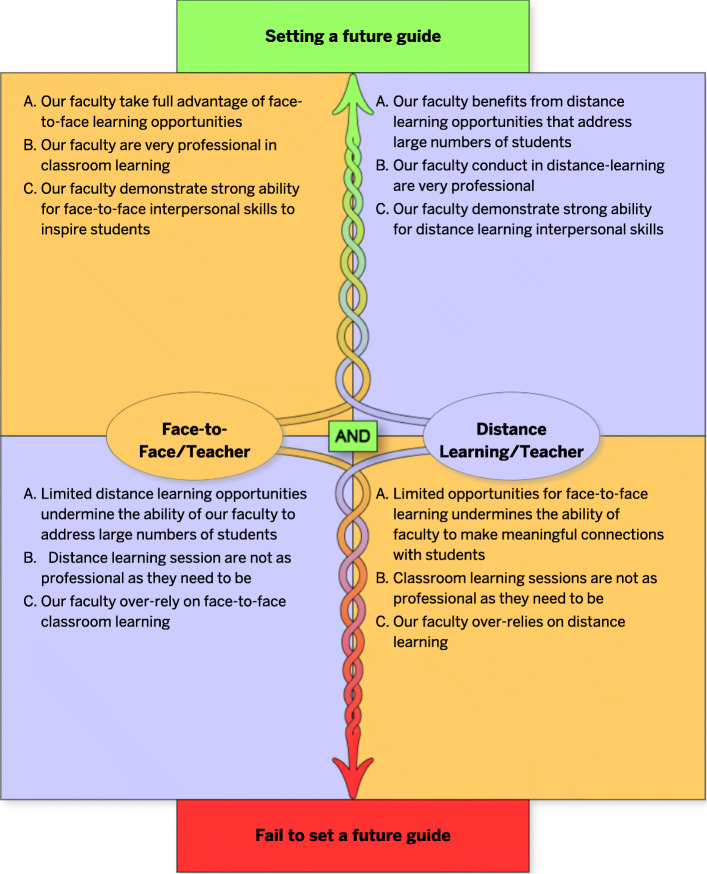
Mapping of the tension; Faculty

A consensus among 134 of the participants that teachers in face to face are almost always professional and demonstrate interpersonal skills to inspire students while the downside of this as 41 participants agree that teachers overfocus on face to face in the classroom teaching. On distance teaching the prominent downside is limited opportunities to make meaningful connections with students as stated by 54 of the respondents (Fig. 2).

### Second tension area: students

Students, in face to face learning appreciate the multiplicity of opportunities they are given without having to deal with technology challenges as stated by 65 participants. On the other hand, special needs students don’t feel at ease attending physically as demonstrated by 46 participants.

**Fig. 3:**
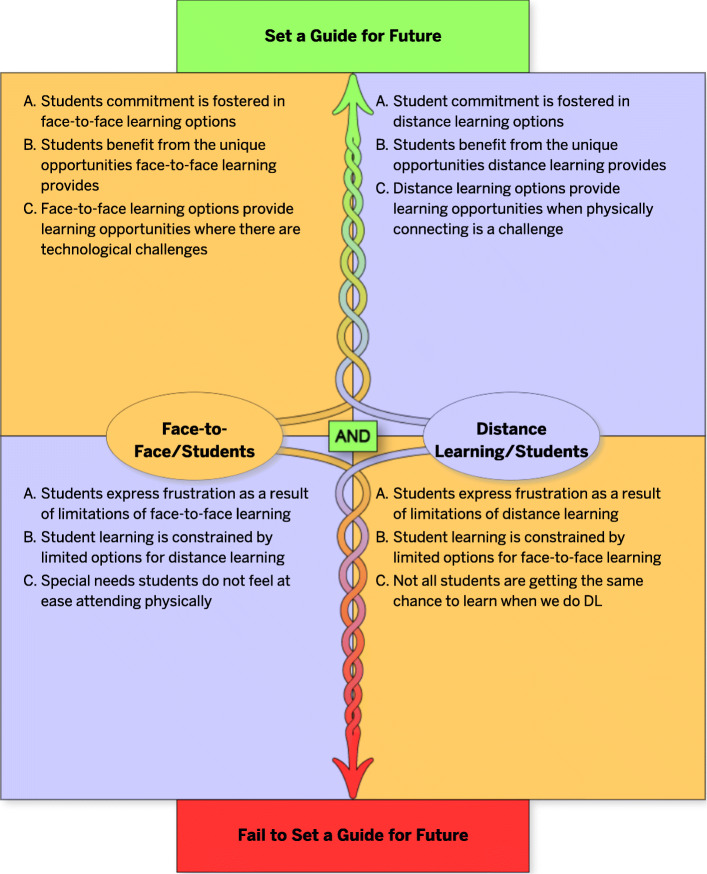
Mapping of the tension; Students

When teaching is at a distance, 64 respondents agreed that this for them represents an opportunity instead of face to face teaching especially in circumstances that might hinder normal teaching. Unfortunately, not all students get the same chance to learn using DL as stated by 48 participants (Fig. 3).

### Third tension area: social issues

The social tension area shows that in face to face almost always the professional relationship with the students is accomplished. This method also apparently promotes teamwork which is on the contrary to distance learning.

**Fig. 4:**
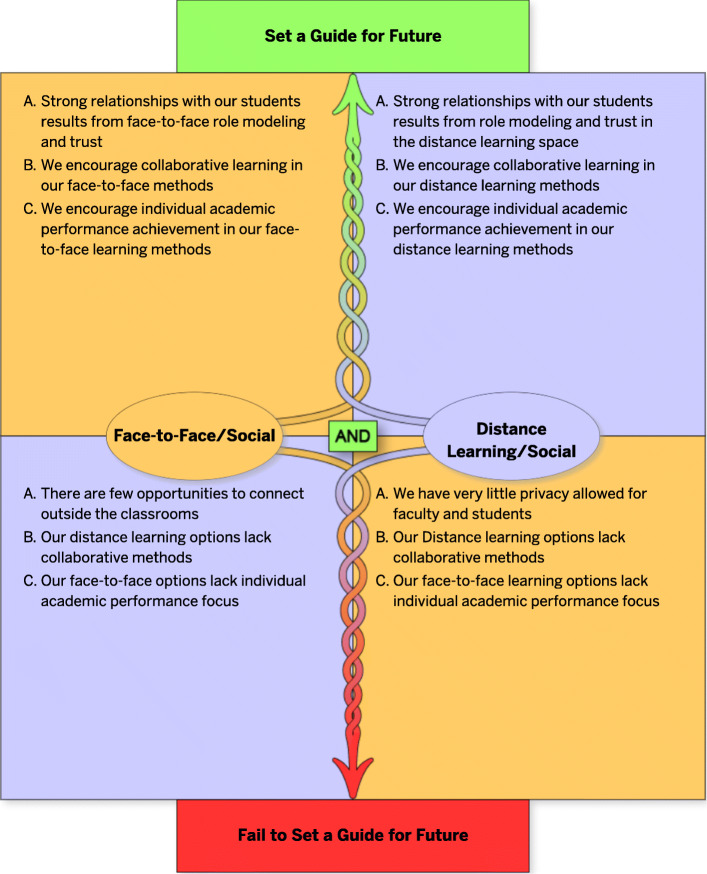
Mapping of the tension; Social

Based on the results of the assessment two areas were marked as high risk. These areas indicate issues that emerge with the use of distant learning in teaching both clinical skills and cognitive communication. These two items scored high in the assessment (around 35 of the respondents highlighted those two items as potentially often to occur) (Fig. 4).

### Fourth tension area: curriculum

**Fig. 5:**
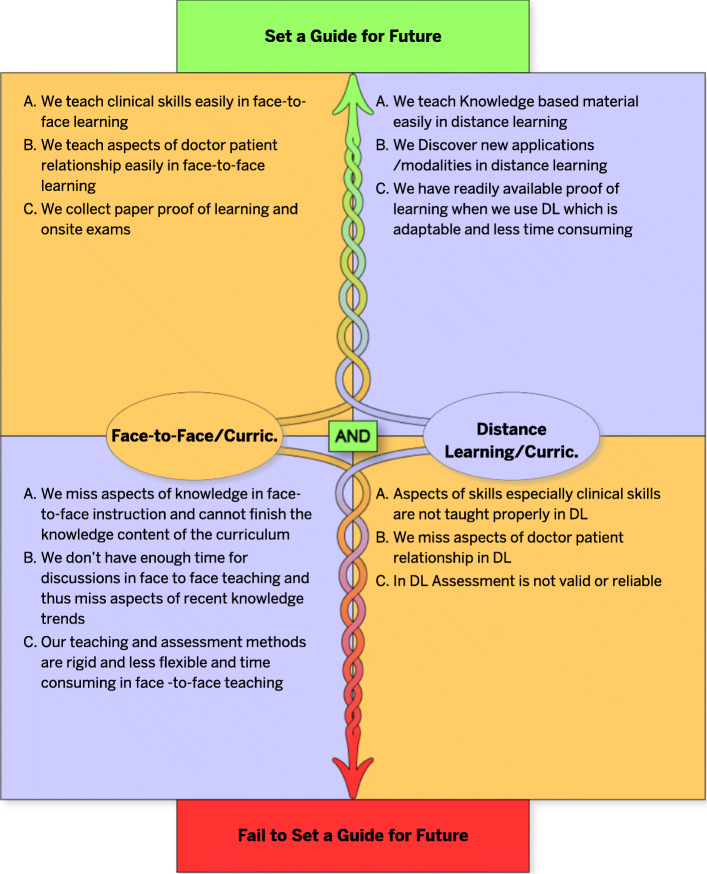
Mapping of the tension; Curriculum

The fourth tension map deals with issues related to the curriculum. In this map it is obvious that there are areas in the curriculum that need to be addressed by face to face interaction including clinical skills and doctor patient relationship as was evident by the high indicated risk associated with both of these items in the downside of the DL pole where 35 of the respondents marked each of these items as often to occur (Fig. 5).

This map also highlighted two potential opportunities in this area of curriculum Components highlighting the importance of use of face to face learning in teaching the above two curriculum elements.

### Fifth tension area: logistics

**Fig. 6:**
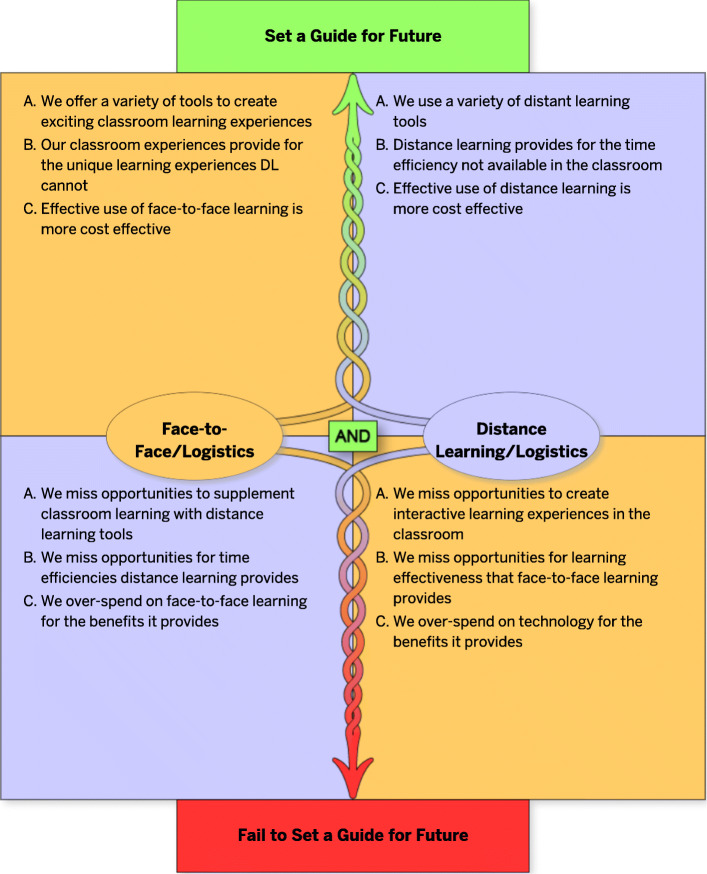
Mapping of the tension; Logistics

A consensus among 59 of the participants that logistics in face to face almost always offer a variety of tools to create an exciting learning classroom meanwhile 46 of the participants agree that logistics we miss the opportunity to supplement the classroom with DL tools. Respondents highlighted the value of time effectiveness of the distance learning as stated by 62 participants. Yet it was noted that distance learning offers less variety in opportunities for learning as stated by 47 of the participants Fig. 6.

#### Processing results to develop an action plan

**Fig. 7:**
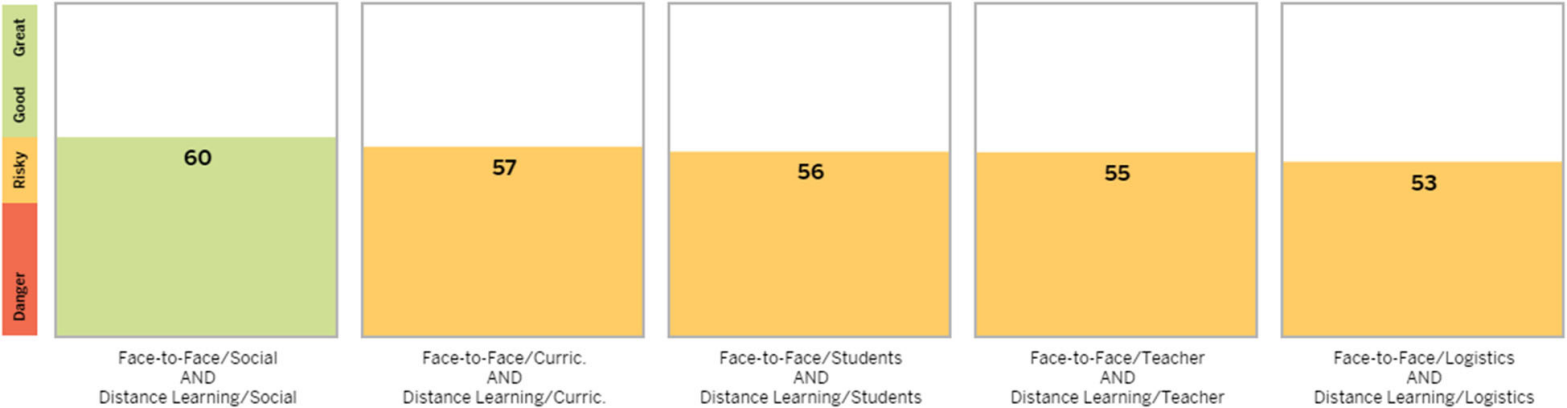
Overall mapping of the five tension areas; Faculty, Students, Curriculum, Social aspects and Logistics

An overall look at the assessment results shows a good leveraging of the tension in the social area only whereas all the other tensions show a failed leveraging state (Fig. 7).

#### Warning signs

Analyzing the data collected from the mapping sessions resulted in identification of a number of warning signs that needed to be included in decision making in any planning process for educational decisions after the COVID-19 era. These were listed in Table 2:

**Table 2:** Warning signs for early avoidance of drawbacks of face to face & distance learning in post COVID-19 era

Warning signs when using face to face teaching	Warning signs when using distance learning
1-When the percentage of face-to-face teaching is more than 70% in a course report 2-30% special needs student do not attend 3-More than 30% of special needs students are dissatisfied with face to face attendance 4-50% of group work is delayed 5-Increase in the incidence of conflicts between students by 10% 6-Decrease attendance of students in group activities by 30% compared to individual work 7-Dissatisfaction and low scores in 30% peer evaluation 8-Dissatisfaction of 20% of students with the quality and up to dateness of offered classroom material	1-Students can identify less than 50% of the names of their teachers in a survey 2-Results of a survey question on student faculty relationship show 40% dissatisfaction of students with faculty engagement. 3-40% of reports of mentorship programs show no significant engagement and report no problems. 4-Failure of 30% of students to submit assignments in DL 5-30% of students fail to attend the synchronous online classrooms 6-30% of students believe that they are at a disadvantage with DL in a student survey question 7-30% decrease in mean scores of formative assessments in DL compared to F2F 8-30% of students and teachers complain about software user-friendliness 9-Students scores in OSCE exams are 10% lower in stations pertaining to topics taught distant than those in stations pertaining to topic taught face to face 10-Increase in violence incidents in the wards and OP related to miscommunication between young physicians and patients by 10% of a 5 year study 11-Lower scores of students on topics taught distant by 10% than topics taught face to face

#### Action steps

Mapping session participants identified a number of action steps that needed to be adopted post COVID-19 era. These are listed in Table 3.

**Table 3:** Action steps to sustain upsides in face to face / distance learning in post COVID-19 era

Action Steps when using face to face learning	Action steps when using distance learning
1-Annual study of percentage of face to face to distant learning in the course report 2-Continuous faculty development (communication skills, presentation skills, mentorship, teaching strategies, student engagement) 3-Strategy for promotion that includes star points on FD courses 4-Continuous appraisal using peer evaluation and CQI 5-Include an evaluation question related to the quality of the relationship with faculty fostered by distance learning and a question about the quality of the student teacher relationship 6-Satisfaction survey annually for students of special needs 7-Increase interactivity of classrooms through (Monitoring of teaching strategy through periodic surveys) 8-Add a survey quarterly for students testing; satisfaction with educational equity 9-Ensure that curricula contain collaborative assignments 10-Peer feedback on group work before delivery of output 11-Teacher of the year nomination 12-Increase social activities (1 annual trip and 2 annual fun days) 13-Continuous monitoring of group activities in classrooms (number, quality efficiency) 14-Increase Clinical skills session performed in F2F to more than 50% of the f2f encounters 15-Encourage f2f communication skills sessions to be a part of the core course requirements 16-Annual analysis of OSCE station results 17-Annual revision of the course report section on teaching methods and issuing a report on the degree of creativity in utilizing existing tools 18-Annual feedback forms containing a question for student about the quality of the tools used in the classrooms	1-Add a survey question to test the acceptability of software used annually 2-Committee responsible for testing and benchmarking applications used for DL and making decisions on what to use 3-Assess hospital incident reports annually and issue a report 4-Activate university auditing systems to ensure proper use of the given tools (quality assurance unit audit) 5-Perform a grade variant analysis annually for student exams and map them topic scores to the teaching method used 6-Set guidelines for attending virtual classrooms and synchronous distance teaching to allow for better utilization of time saved on commuting Faculty development program (technical) 7-Survey of available special needs facilities in the university 8-Monitor analytics quarterly for: attendance rate, assignment submission, mean scores in formative exams 9-Continuous maintenance and improvement of LMS, 10-Back up learning resources

## Discussion

### Teacher tension

The concept of professionalism is embedded into our teaching practice and is inherently built up in practice as we develop. Teachers have been taught to monitor their professional behaviour and this is a construct that refers them to a number of acceptable behaviors that are within the educational culture norms. These acceptable behaviors have developed very little since their dawn and change in the educational platforms require that we revisit the core concept of professionalism. Tested against the test of time and technology, these norms are challenged every day and need to be addressed and updated to accommodate for new educational limitations and challenges [10].

Creating meaningful connections with students requires that we develop new ways to build virtual communities. Students often use social media to interact and share information with each other, but they can feel uncomfortable having their professor in this space. This calls for new norms that need to be adopted (the connection.com) [11].

### Student and social tension

Sajid et al., 2016 [12], Papanna et al., 2013 [13], and Murphy 2020 [14] reported that the flexibility of asynchronous online lectures as a component of DL allow students to pace themselves individually and eliminate the barriers of time, location, personal or financial circumstances. Longhurst et al., 2020 [15] reported reduced student engagement as an environmental threat in 36% of universities in their SWOT analysis from 14 different universities in the United Kingdom and Republic of Ireland. This is in agreement with the findings in this study.

Distance learning must engage, guide and retain the student. In opposition to current dominant ideas about self-directed learning, distance learning works best when students are absolutely clear about the pathway that they must follow. Giving students options and choices at a distance is problematic. Distance learning students can get lost. They tend not to have a consistent or predictable approach to using such options which means that the distance teacher cannot be sure about what is being learned, unless the pathway is clear and specified [7].

Longhurst et al., 2020 [15] discussed the issue of teacher student relationship where 21% of the universities participating set off an alarm that student teacher relationship is an environmental threat they are facing.

### Curriculum and logistics tension

Warning signs regarding clinical teachingin this study are in agreement with Longhurst et al. 2020 [15] who expressed that 50% of participating universities highlighted Lack of practical sessions and labeled thist as a weakness point. Also in agreement with Papanna et al., 2013 [13] who emphasized the importance of the orientation classes to students fresh to clinics in helping them understand the bedside clinics better and the other barriers identified by the students’ needs to be addressed by the management.

Nuffer and Duke (2013) [16] found that In the final year of a Doctor of Pharmacy program, live training was more effective than online training in preparing students to become oriented to diabetes management clinic operations and for students to have confidence seeing patients early in the rotation. This finding is similar to opinions that persist to this day and were evident in our mapped results.

In our study a need emerged for combining DL with other forms of education. This is in harmony with Reyna 2020 to use flipped learning as friendly learning design to develop lifelong learning skills [17].

This demonstrates a better understanding of the concept of distance learning in its described definition in Grant 2008 [9]. This form when best applied and executed will in itself be a hybrid form of education that allows for proper engagement and follow up of students. In DL learners can be lost when there is too much emphasis on self-directed learning. This led experts to renown self-directed learning when describing best DL practices [7].

Distance learning is a whole system which integrates a wide variety of elements for curriculum coverage. These might include print, use of resources, practical classes, technology-based methods, face-to-face learning, individual and group work and many others [7].

### Warning signs and action steps

From the identified steps, in the process of delivering the educational services, it seems of extreme importance that schools, and institutions focus on introducing and applying quality procedures since quality management routines have become an integral part of the global management structures of higher education institutions [18] & [19]. The cross-cultural gaps and institutional discrepancies have to be taken in consideration when applying the standard measures of quality standards in higher education ensuring near-zero errors in quality management [20].

Vlachopoulos (2020) [21] emphasized the importance of evaluation and monitoring of these New learning initiatives and environments to provide an evidence base to implement online education. This can be through internal and external evaluations.

Internal evaluations follow ups and assessments key driving practices for improvement of the faculty’s internal educational services. It is a necessity that internal quality procedures and focused questionnaire items are monitored closely all around the year to act as triggers for corrective action decisions. In addition, quality unit teams who are responsible for internal quality audits must liaise and work in alignment with the various stakeholders for adapted quality management [22]. Shared knowledge of the academic faculty members as well as the administrative personnel would help the internal quality team develop a benchmark to enhance the internal performance targets for the upcoming years. ‘Faculty autonomy’ is another factor that encourages faculty members to innovate in their research and teaching, sharing their individual thoughts and knowledge with the internal stakeholders [23].

Stensaker et al., 2011 [24] emphasized that external quality assurance had a significant impact on higher education institutions which aroused the need of these institutions to accommodate for these external expectations in terms of response to the quality concerns. Developing quality control, necessitates strengthening the managerial control which according to Brennan & Shah, 2000 [25] could be achieved by developing internal quality management systems directed to governing the educational provision through establishing more recognized organizational rules and routines. Compliance driven quality assurance is another factor that compels higher education institutions to share their knowledge with external interested parties like government agencies in order to develop a benchmark for the rest of the faculties and institutions.

According to Shah 2012 [26], striving to improve the quality assurance in the universities is primarily based in the board agreements and the synergy between the external quality audits and the internal university procedures The ultimate objectives for these quality systems is to enhance the students’ learning experience, through coordination and developing good quality indicators, by following robust Leadership and management strategies learning experience [27].

In their study Longhurst et al., 2020 [14], tested strength points in universities and identified that 71% of the universities consider DL as one of their strength points; this is in contrast to our self-reported findings. This might be attributed to technological advancement in universities in his study and their possession of a well-established LMS.

Also Longhurst et al., 2020 [14] found that time constraints was a weak organizational point in 57% of the universities in DL which is opposing our finding. This can be explained that the perspective of time was different as in our study we were highlighting classroom efficiency while Longhurst’s study discussed time frames in general.

Distance lectures allow students to pace themselves individually and eliminate the barriers of time and location [12] which describes the exact situation in our findings.

The identified warning signs also focus on signs that are relevant to practice like violence incident reports of teaching hospitals and the importance of these reports to stimulate decision to change educational courses and add more face to face interaction.

Content of the curriculum will be shaped by the health needs of the changing population, new medical knowledge and the possible changing role of the doctor from patient advocate to health resource manager [28].

Information for which change, and adaptation decisions are made can be deduced from practice and the incidental opportunities that arise in everyday physician patient encounters. To cope with the rapid pace, the curriculum will have to be a dynamic concept in itself and no single curriculum is likely to produce the variety of doctors we require to meet changing societal needs. Not only does the curriculum need to be flexible, it also needs to adapt across the differing needs of the communities doctors will serve. This will offer learners more choice.

A number of data and valuable information is retrievable from OSCE station results as per the identified warning signs. This puts great emphasis on the analysis of results of OSCE in an analytical way in order to retrieve information usable for curriculum reform.

Evidence based medicine and evidence based medical education is now the baseline accepted practice. Many interventions have been conducted to validate the use of formative OSCE to guide curriculum decisions [29]. Information from OSCE stations has for a long time been used to guide student learning.

## Conclusion

The planning for what comes after COVID-19 needs focus throughout this transition period. Life as we once knew it will no longer be the same. We need to plan for the future with all the necessary adjustments. In order to make this plan as realistic as possible, we need to understand the dynamics of education within the context of polarities. The continuous shift between educational decisions is the standard accepted dynamic. Best practice in this area lies in leveraging this transition. Educators need to understand that the choice of distance learning and face to face learning, although was imposed as a no-alternative solution during the COVID era, yet it has always existed as a possible alternative and will continue to exist after this era. The value of polarity mapping and leveraging allows for opportunities like teacher student social interaction, to be built upon.

On the other spectrum, making shifts in educational decisions between face to face and distance learning in the future requires focus in a set of warning signs that potentially highlight risk before it occurs. Most of these warning signs are indicators extracted from internal quality reports measuring student satisfaction and open ended comments reflecting on student engagement on the educational and social level.

## Data Availability

Model for Utilizing Distance Learning post COVID-19 using (PACT)™ Data set are available at Harvard Dataverse. doi:10.7910/DVN/67CIZB

## Abbreviations

PACT™ model: The Polarity Approach to Continuity and Transformation
DL: Distance learning
